# Knowledge and practice of airway pressure release ventilation in patients with acute respiratory distress syndrome among ICU nurses and respiratory therapists in China: a multicenter cross-sectional survey

**DOI:** 10.3389/fmed.2026.1879189

**Published:** 2026-07-20

**Authors:** Dan Wen, Simei Wang, Xiaoli Du, Yang Hu, Yao Wang, Zhenghua Liang, Qiuyu Liu

**Affiliations:** Intensive Care Unit, Mianyang Central Hospital, School of Medicine, University of Electronic Science and Technology of China, Mianyang, Sichuan, China

**Keywords:** critical care nursing, intensive care units, respiration, artificial, respiratory distress syndrome, respiratory therapists

## Abstract

**Purpose:**

To assess intensive care unit (ICU) nurses’ and respiratory therapists’ knowledge and practice regarding airway pressure release ventilation (APRV) use in acute respiratory distress syndrome (ARDS) patients and identify barriers to its implementation in China.

**Methods:**

A multicenter cross-sectional survey. Between June 22, 2024, and August 10, 2024, an online cross-sectional survey of ICU nurses and respiratory therapists from five cities in Sichuan Province, China, was conducted. Descriptive statistics were used to analyze the data.

**Results:**

Among 239 valid responses from ICU nurses and respiratory therapists in Sichuan Province, 55.2% (132/239) respondents reported no prior didactic APRV training, and 54.4% (130/239) had never used APRV for ARDS. While 97.9% (234/239) correctly identified ARDS as an indication, substantial variability was observed in the alignment of parameter settings with evidence-based recommendations. Key barriers to APRV use included lack of guidelines/standardized protocols (56.5%, 135/239), insufficient training (57.3%, 137/239), and lack of awareness (45.2%, 108/239).

**Conclusion:**

Considerable variability in APRV knowledge and practice was observed among ICU nurses and respiratory therapists, coupled with a lack of standardized training and institutional protocols, highlighting a critical need for targeted educational interventions. The lack of guidelines or standardized protocols is the most common impediment to APRV use.

## 1 Background

Airway pressure release ventilation (APRV) is a mode of ventilation that involves continuous positive airway pressure (CPAP) ventilation with periodic transient pressure release. It is often used in the management of patients with acute respiratory distress syndrome (ARDS) and hypoxemia (1). In 1987, Stock and Downs (2) first described APRV as a mode of ventilation based on the concept of “open lung,” which improves lung oxygenation and ventilation by maintaining CPAP with intermittent pressure release while avoiding alveolar hyperinflation or collapse. ARDS is a common critical respiratory condition, with mortality rates of patients with severe ARDS ranging from 40% to 60% (3). The lung- protective ventilation strategy, commonly used for the clinical treatment of ARDS, combines two techniques: low tidal volume ventilation (LTV) and positive end-expiratory pressure (PEEP) ventilation (4). The application of LTV aims to prevent pneumatic pressure injuries due to alveolar hyperinflation, thereby protecting the lungs from mechanical damage (5). The use of PEEP can effectively reduce the occurrence of pulmonary atelectasis and promote alveolar re-expansion and gas exchange, thus improving respiratory function in patients (6).

Airway pressure release ventilation is a time-periodic, pressure-controlled mode of intermittent mandatory ventilation with a high inspiration-to-expiration (I:E) ratio. It helps improve oxygenation through sustained high pressure to keep the alveoli open and provide transient releases to eliminate carbon dioxide and prevent alveolar collapse. A retrospective analysis by Yoshida found an increase in the P/F (PaO_2_/FiO_2_) ratio and the percentage of lung ventilation in patients treated with APRV (7). Simultaneously, APRV allows unrestricted voluntary breathing throughout respiration, potentially reducing the need for sedation (8–10). Putensen (11) found that autonomic ventilation enhances comfort in patients with ARDS, thereby reducing the need for sedative medication. This change was directly associated with a significant reduction in the number of ventilator days (*p* = 0.011) and length of intensive care unit (ICU) stay (*p* = 0.032) for patients in the APRV group. In addition, APRV prevents ventilator-associated lung injury by maintaining alveolar epithelial integrity and alveolar surface-active substances to maintain alveolar stability (1).

Precise control of APRV parameter settings is essential for improving the patient’s ventilatory function and oxygenation status, including the parameters of high pressure (P-high), low pressure (P-low), high time (T-high), and low time (T-low) (1). P-high and T-high are determinants of airway pressure that can affect oxygenation. By adjusting the P-high and P-low levels, the APRV can maintain alveolar stability. Currently, two primary parameter-setting strategies exist for implementing APRV. In fixed setting APRV, the setting of the pressure release phase is constant; in other words, the value of the release pressure remains fixed regardless of changes in the patient’s pulmonary respiratory mechanics. The personal setting of the APRV is dynamically adjusted to the pressure-release phase according to the patient’s pulmonary respiratory mechanics, particularly the change in the slope of the expiratory flow curve. This personalized approach can be more precisely adjusted to meet patient’s specific needs, thereby reducing the risk of lung injury (12). However, patients who breathe independently during P-high may be subjected to large cross-lung pressures and tidal volume (TV) that exceed recommended limits (13). Therefore, patient-ventilator interactions must be monitored closely by experienced clinicians to prevent harm, especially in healthcare organizations where APRV use is still in its early stages.

Despite the potential advantages of APRV, questions regarding its safety continue to concern clinicians and researchers because of its variability and the fact that clinicians are not familiar with it. Surveys in Saudi Arabia (14, 15) have shown that most nurses working in the ICU have not been trained adequately in the APRV mode, and there is a lack of consensus among physicians regarding the use of the APRV mode. However, it is not widely known how Chinese ICU caregivers perceive and apply APRV to patients with ARDS. Therefore, this study conducted surveys in several cities in Sichuan Province, China, which aimed to assess the knowledge and practice of using the APRV mode for patients with ARDS and to identify the barriers of using APRV among nurses who work in the ICU.

## 2 Aim

To assess ICU nurses’ and respiratory therapists’ knowledge and practice regarding APRV use in ARDS patients and identify barriers to its implementation in Sichuan Province, China.

## 3 Materials and methods

### 3.1 Study design

A cross-sectional design was used to survey ICU nurses and respiratory therapists from five cities in Sichuan Province, China, from June 22, 2024, to August 10, 2024. ICU nurses and respiratory therapists were enrolled as study participants. The inclusion criteria included direct involvement in medical care in the ICU, at least 1 year of clinical experience, informed consent, and voluntary participation in this study. The exclusion criteria included those not directly involved in the ICU and those who refused to participate in this study. This study was conducted in accordance with the principles of the Declaration of Helsinki. This study was approved by the Ethics Committee of Mianyang Central Hospital (Approval no. S20240233-02) (approved on 21 June 2024). Based on Kendall’s sample size estimation principle, the required sample size was estimated to be 5–10 times the number of questionnaire items. With 26 items in the questionnaire and accounting for a 20% rate of invalid responses, the target sample size was calculated to be between 156 and 312 participants.

### 3.2 Questionnaire design

The questionnaire was adapted with permission from Abdulelah (14), which was initially applied to investigate how nurses working in critical care units in Saudi Arabia used the APRV mode to manage patients with ARDS. To ensure cultural relevance and content validity for the Chinese healthcare context, a rigorous modification process was undertaken.

The original English questionnaire was translated into Chinese following the forward-backward translation method. Two bilingual experts in critical care (one ICU physician and one senior nurse specialist) independently translated the questionnaire into Chinese. Discrepancies were resolved through a consensus meeting. Subsequently, a separate bilingual expert, blinded to the original version, back-translated the Chinese version into English. The research team compared the back-translated version with the original to ensure conceptual equivalence rather than just linguistic accuracy.

The Chinese draft was then reviewed by a multidisciplinary expert panel consisting of three ICU physicians, two respiratory therapists, four senior ICU nurses (with over 10 years of experience), and two nursing graduate students specializing in critical care. These experts evaluated the questionnaire for clarity, relevance, and comprehensiveness regarding APRV knowledge and practice in China. The Item-level Content Validity Index (I-CVI) and Scale-level Content Validity Index (S-CVI) were informally assessed based on expert feedback; items with ambiguity or low relevance were revised or removed.

Once the final draft was completed, a pilot test was conducted with 10 ICU nurses who met the inclusion criteria but were not part of the main study. These nurses were asked to complete the questionnaire and provide feedback on item clarity, structure, and estimated completion time. Based on their feedback, minor wording adjustments were made to improve comprehensibility. The pilot data were excluded from the final analysis.

The final questionnaire contained four sections. Part I: General information, including gender, age, occupation, professional title, educational background, years of experience, whether the individual is a specialist nurse, participation in ventilator training, experience using APRV in patients with ARDS, and whether they have received didactic training in APRV ventilation mode. Part II: Previously relevant literature on APRV was referred to for setting, and the survey included both indications and parameter settings of APRV. (1) Indications for APRV, including conditions for using APRV modalities and strategies to improve oxygenation when conventional mechanical ventilation fails. (2) APRV parameter settings, including P-high, P-low, T-high, and T-low settings; maximum permissible TV for patients with ARDS; maximum permissible settings for P-high; and utilization of pressure support during spontaneous breathing. Part III: APRV weaning, including criteria for withdrawing P-high, withdrawing T-high, and switching ventilation mode to CPAP. Part IV: Barriers to the use of the APRV mode which aims to understand the factors hindering the application of the APRV mode in clinical practice.

### 3.3 Data collection

Online questionnaires were distributed and collected using the Wenjuanxing software. Before the survey, the study members contacted the head nurses of the ICU in the hospitals where the survey was conducted to inform them of the purpose and content of the survey and obtained consent to release the link to the questionnaire and the quick response (QR) code. The questionnaire included an informed consent form and standardized guidelines that explained the purpose of the survey, its content, and the precautions to be taken. To ensure the quality of the questionnaire, it was filled out anonymously, and all options were mandatory and could only be submitted after completion.

### 3.4 Data analysis

Data were analyzed using descriptive statistics. The results are summarized using frequencies and percentages. Statistical analyses were performed using the SPSS 26.0 statistical software for Windows (SPSS, Chicago, IL, USA).

## 4 Results

### 4.1 Demographic information

In total, 270 questionnaires were distributed in this study, and 239 questionnaires were validly recovered, with a valid recovery rate of 88.52%. 25.5% (61/239) respondents were men and 74.5% (178/239) were women. In total, 67.4% (161/239) were non-specialist nurses, 13.8% (33/239) were respiratory therapists, 56.5% (135/239) had received ventilator training, 55.2% (132/239) were not trained in APRV mechanical ventilation mode, and 54.4% (130/239) of respondents were not trained in the use of APRV for with ARDS patients. Details are presented in Table 1.

**TABLE 1:** Demographic characteristics of ICU nurses and respiratory therapists (*n* = 239).

Demographics	*n* (%)
Gender	
Men	61 (25.5%)
Women	178 (74.5%)
Age	
20∼30	111 (46.4%)
31∼40	99 (41.4%)
41∼50	26 (10.9%)
≥51	3 (1.3%)
Occupation	
Respiratory therapist	33 (13.8%)
Nurse	206 (86.2%)
Professional title	
Junior professional title	121 (50.6%)
Intermediate professional title	101 (42.3%)
Senior professional title	17 (7.1%)
Education	
Junior college	62 (25.9%)
Bachelor’s degree	174 (72.8%)
Master’s degree and above	3 (1.3%)
Years of clinical experience	
≤5 years	76 (31.8%)
6∼10 years	76 (31.8%)
11∼15 years	47 (19.7%)
≥16 years	40 (16.7%)
Specialist nurse	
No	161 (67.4%)
Intensive care specialist nurses	49 (20.5%)
Respiratory therapy specialist nurse	12 (5.0%)
Other specialist nurses	17 (7.1%)
Received training on ventilator	
Out-of-hospital training	31 (13.0%)
In-hospital training	55 (23.0%)
Departmental training	135 (56.5%)
Online self-study	6 (2.5%)
Have not attended training	12 (5.0%)
Used APRV mode in ARDS patients	
Yes	109 (45.6%)
No	130 (54.4%)
Received training on APRV mode	
Yes	107 (44.8%)
No	132 (55.2%)

### 4.2 Indication of APRV and improvement strategy of ARDS oxygenation

**FIGURE 1:**
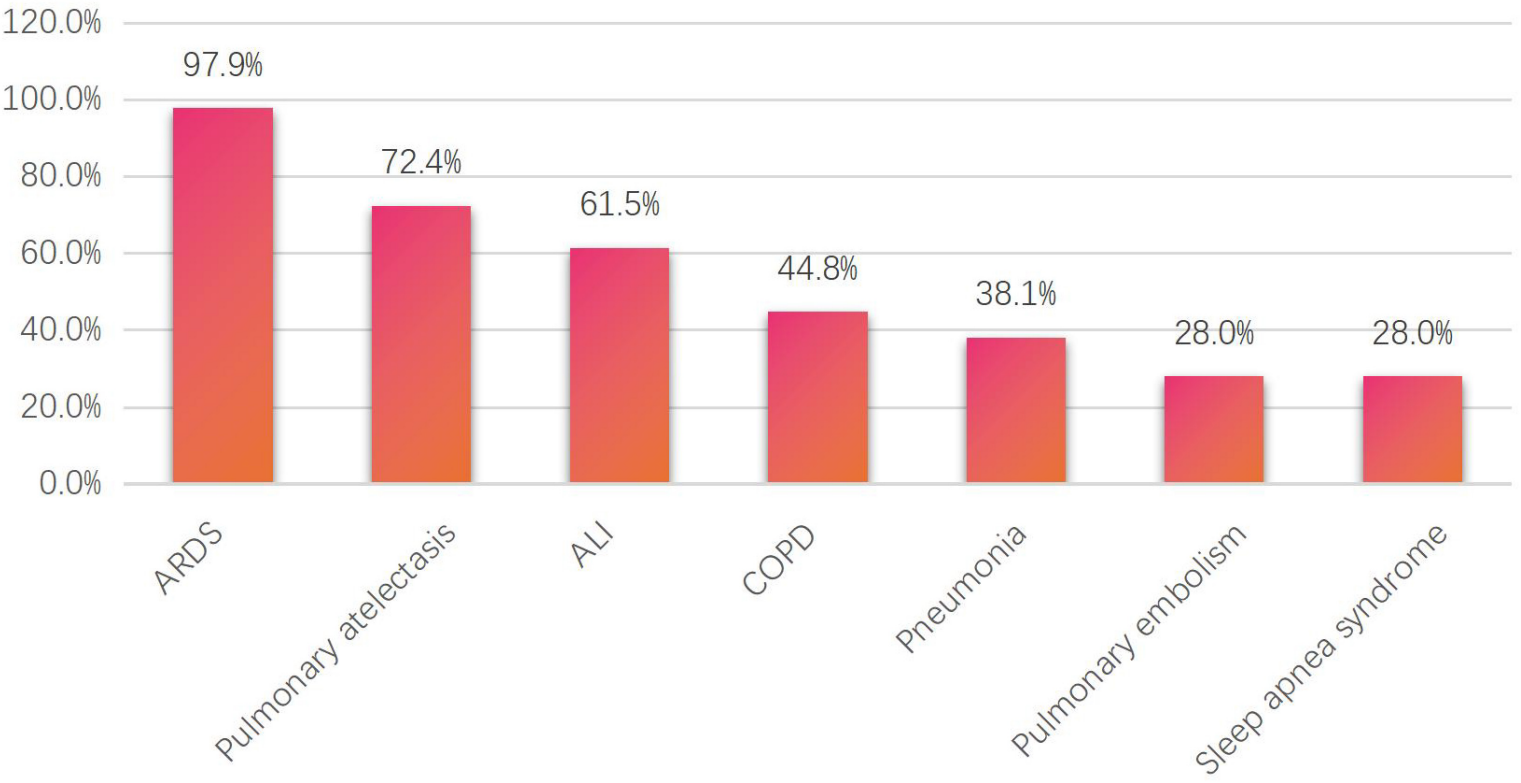
Indications for APRV. APRV, airway pressure release ventilation; ARDS, acute respiratory distress; ALI, acute lung injury; COPD, chronic obstructive pulmonary disease.

**FIGURE 2:**
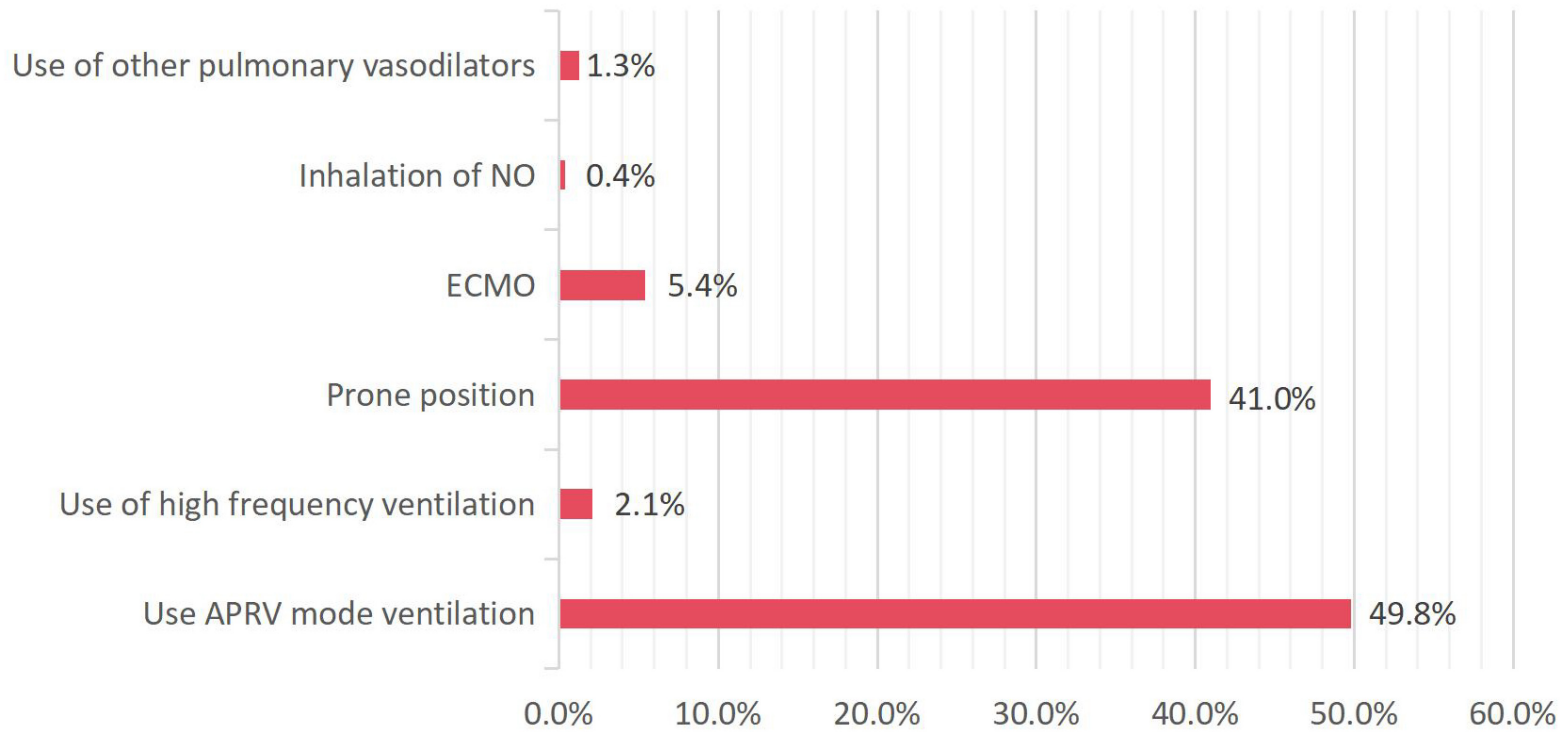
Strategies to improve oxygenation in ARDS. ARDS, acute respiratory distress; NO, nitric oxide; ECMO, extracorporeal membrane oxygenation.

Approximately 97.9% (234/239) of respondents identified ARDS as an indication for APRV, followed by pulmonary atelectasis (72.4%, 173/239), acute lung injury (ALI) (61.5%, 147/239), chronic obstructive pulmonary disease (COPD) (44.8%, 107/239), pneumonia (38.1%, 91/239), pulmonary embolism (28%, 67/239), and sleep apnea syndrome (28%, 67/239). The details are presented in Figure 1. When traditional mechanical ventilation modes failed to improve oxygenation in patients with ARDS, 49.8% (119/239) respondents selected APRV as a strategy to improve oxygenation in patients with ARDS. The details are presented in Figure 2.

### 4.3 Parameter setting of APRV

Regarding the initiation of APRV for patients with ARDS, only 16.3% (39/239) of respondents selected the evidence-based correct initial P-high setting (equal to the plateau pressure of a conventional ventilator). For P-low, 15.5% (37/239) correctly identified 0 cmH_2_O as the recommended initial setting. Regarding the T-high setting, 33.1% (79/239) chose the consensus-recommended initial duration of 4–6 s. For T-low, the highest proportion of respondents (12.1%, 29/239) selected the correct criterion of setting the expiratory flow rate at 50%–75% of the peak expiratory flow rate (PEFR). In terms of safety limits, 38.1% (91/239) correctly identified 4–6 ml/kg as the maximum permissible tidal volume during the release phase. Regarding the upper limit of P-high, 32.2% (77/239) selected the recommended maximum of 35 cmH_2_O. Finally, 74.1% (177/239) correctly affirmed the use of pressure support during spontaneous breathing in APRV mode. Details are presented in Table 2.

**TABLE 2:** Parameter setting of APRV (*n* = 239).

Variables	*n* (%)
Initial P-high setting	
25 cmH_2_O	53 (22.2%)
Equal to the plateau pressure of a conventional ventilator	39 (16.3%)
Equal to the average airway pressure of a conventional ventilator	21 (8.8%)
Mean airway pressure 2∼5 cmH_2_O higher than conventional ventilators	81 (33.9%)
Achieve a tidal volume of 6 ml/kg/pbw (projected body weight)	45 (18.8%)
Initial P-low setting	
0 cmH_2_O	37 (15.5%)
2∼5 cmH_2_O	89 (37.2%)
Matching PEEP with conventional ventilators	48 (20.1%)
Variable depending on oxygenation	65 (27.2%)
Initial T-high setting	
2∼3 s	30 (12.6%)
4∼6 s	79 (33.1%)
Required ventilation and respiratory rate per minute	76 (31.8%)
Inhalation to exhalation (I: E) ratio per inhalation	54 (22.6%)
Initial T-low setting	
Setting time (0.4∼0.8 s)	73 (30.5%)
Based on the desired inspiratory to expiratory (I: E) ratio	63 (26.4%)
When expiratory flow rate is equal to 25%∼49% of peak expiratory flow rate	74 (31.0%)
When expiratory flow rate is equal to 50%∼75% of peak expiratory flow rate	29 (12.1%)
Maximum allowed tidal volume	
4∼6 ml/kg	91 (38.1%)
7∼8 ml/kg	87 (36.4%)
9∼10 ml/kg	37 (15.5%)
>10 ml/kg	18 (7.5%)
No limits	6 (2.5%)
Maximum allowed P-high	
30 cmH_2_O	102 (42.7%)
35 cmH_2_O	77 (32.2%)
40 cmH_2_O	44 (18.4%)
No maximum	16 (6.7%)
Pressure support utilization during spontaneous breathing	
Yes	177 (74.1%)
No	62 (25.9%)

### 4.4 APRV weaning and discontinuation

During the weaning process, 37.7% (90/239) of respondents correctly identified the criterion of gradually reducing P-high to a target of 20 cmH_2_O. For T-high weaning, 38.5% (92/239) selected the correct approach of gradually increasing it to 10 s. Regarding the transition to CPAP, the vast majority of respondents (74.5%, 178/239) correctly recognized that switching is appropriate only when all criteria are met: FiO_2_ ≤ 40%, P-high ≤ 10 cmH_2_O, and T-high ≥ 10 s. Details are presented in Table 3.

**TABLE 3:** Airway pressure release ventilation (APRV) weaning and discontinuation.

Variables	*n* (%)
Which of the following criteria should be used to wean P-high during APRV ventilation mode in patients with ARDS?	
Gradually reduce the P-high and gradually reach the target of 20 cmH_2_O	90 (37.7%)
Gradually reduce the P-high and gradually reach the target of 15 cmH_2_O	53 (22.2%)
Gradually reduce the P-high and gradually reach the target of 10 cmH_2_O	63 (26.4%)
Gradually reduce the P-high and gradually reach the target of 5 cmH_2_O	33 (13.8%)
Which of the following criteria should be used to wean T-high during APRV ventilation mode in patients with ARDS?	
Gradually increase the T-high to gradually reach the target of 7 s	66 (27.6%)
Gradually increase the T-high to gradually reach the target of 10 s	92 (38.5%)
Gradually increase the T-high to gradually reach the target of 15 s	60 (25.1%)
Gradually increase the T-high to gradually reach the target of 20 s	21 (8.8%)
When a patient with ARDS achieves oxygenation goals and is stabilized, the mode of ventilation should be switched to CPAP based on which of the following criteria?	
FiO_2_ ≤ 40%	20 (8.4%)
P-high ≤ 10 cmH_2_O	30 (12.6%)
T-high ≥ 10 s	11 (4.6%)
All criteria mentioned above (FiO_2_ ≤ 40%, P-high ≤ 10 cmH_2_O, T-high ≥ 10 s)	178 (74.5%)

### 4.5 Barriers to the use of the APRV mode

**FIGURE 3:**
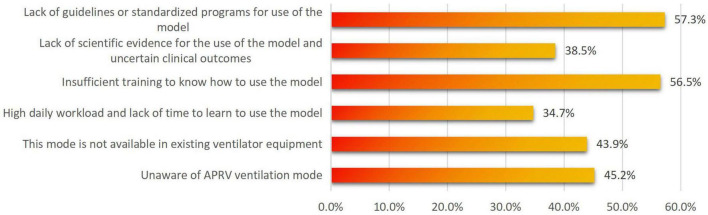
Barriers to the use of the APRV mode. APRV, airway pressure release ventilation.

The top three factors that hindered the use of APRV modalities in daily practice from the healthcare worker’s perspective were lack of guidelines or standardized protocols for their use (56.5%, 135/239), insufficient training (57.3%, 137/239), and unawareness of APRV modalities (45.2%, 108/239). The details are presented in Figure 3.

## 5 Discussion

Our survey revealed substantial variability in the awareness and application of the APRV mode among ICU nurses and respiratory therapists caring for patients with ARDS. The APRV mode is underutilized in patients with ARDS, and most nurses are not trained in APRV. In addition, talent resources for respiratory therapy are scarce.

The findings of this study showed that most respondents (56.5%) were not adequately trained of APRV, although they had received ventilator training in the unit. This is because most nurses (55.2%) had not received didactic training on APRV, which resulted in their lack of awareness of APRV, consistent with the findings of Aldhahir (14, 15). A total of 54.4% of the nurses had never used APRV in patients with ARDS. This shows that the clinical application of APRV by nurses in the ICU is inadequate and lacks practical application in patients with ARDS. The findings of a study by Sadowitz (16) showed that the appropriate use of APRV is the simplest and most feasible way of preventing the onset and progression of ARDS. Therefore, to enhance the knowledge of ICU healthcare professionals on APRV and to promote its application in patients with ARDS, specialized training on APRV should be actively implemented to ensure timely updates to the knowledge base. Respiratory therapists, personnel trained in specialized mechanical ventilation, have an advantage in APRV mode selection, parameter setting, monitoring, and evaluation. Only 13.8% of the respondents to this survey were respiratory therapists, and there was a serious shortage of respiratory therapists in China. Active academic education, in-service training, and professional certification by respiratory therapists are the ways forward.

In our study, 97.9% of caregivers considered ARDS as the primary indication for APRV, which is consistent with the findings of Camporota (17). However, the indications for APRV, the effectiveness of its application in patients with ARDS, parameterization, and management are still controversial (1, 18–20). Larger, high-quality studies are needed in the future to determine their effectiveness and safety, and to develop standardized, regulated management protocols. However, APRV has certain advantages in the treatment of hypoxemic patients with ARDS, and by selecting and adjusting its important parameters, it can effectively promote alveolar re-expansion and gas exchange in patients with ARDS (15).

The findings of our study revealed that 49.8% of caregivers would choose to use APRV mode to improve oxygenation in patients with ARDS when traditional mechanical ventilation modes fail, which is consistent with the findings of Daoud (21). It has been shown (22) that early application of APRV in patients with ARDS reduces ventilation time and length of ICU stay and improves oxygenation outcomes. A Meta-analysis (23) discussed the efficacy of APRV in the treatment of ARDS and found that APRV increased the PaO_2_/FiO_2_ ratio in patients with ARDS, thereby improving their oxygenation status and clinical symptoms. Current strategies for improving oxygenation in patients with ARDS include pharmacological intervention strategies, such as inhaled nitric oxide and inhaled prostacyclin. A higher PEEP can be used in patients with moderate-to-severe ARDS, and veno-venous extracorporeal membrane oxygenation can improve oxygenation in patients with severe ARDS (24–26).

The prone position has been shown to cause redistribution of alveolar gases, reduce shunting, decrease lung strain, and improve ventilation in patients with ARDS, and has long been a standard treatment procedure for ARDS (27). Guidelines strongly recommend using the prone position to improve ventilation in patients with ARDS (24, 25). This study result shows that approximately 41% of the caregivers would choose the prone position to improve the oxygenation status of ARDS patients, as illustrated in Figure 2. Ventilation in the prone position reduces the compression of the lungs on the alveoli in the gravity-dependent zone close to the spinal side, reduces the intrathoracic pressure in the gravity-dependent zone, and results in an even distribution of the intrathoracic pressure on the ventral and dorsal sides of the pleural cavity. It also reduces compression of the heart and mediastinum on some lung tissues, facilitating partially collapsed dorsal alveolar re-expansion and improving ventilation in the dorsal region (28). Therefore, prone position ventilation significantly reduces the intrapulmonary shunt in the dorsal region without increasing the intrapulmonary shunt in the ventral region, which reduces the intrapulmonary shunt and thus improves oxygenation in patients with ARDS (29).

Only 16.3% of the nurses in our study believed that P-high should be consistent with the measured plateau pressure, and some studies have suggested aligning P-high with plateau pressure in volume-controlled mode or inspiratory pressure in pressure-controlled mode. Also, 15.5% believed that P-low should be set to 0 cmH_2_O, and because APRV produces spontaneous PEEP, P-low could be set to 0 cmH_2_O to reduce convective expiratory airflow resistance and maximize ventilation. There have also been studies in which P-low was set to physiological PEEP (5 cmH_2_O), and minimal pressure was used to prevent atelectasis according to the patient’s condition. A total of 32% of survey respondents felt that T-high should start between 4 and 6 s, which is consistent with the APRV protocol. T-high is usually set to 90% of the total cycle time per breath. Approximately 26.4% of survey respondents believed that for patients with ARDS, T-low should be adjusted for the required inspiratory-to-expiratory (I:E) ratio. And 31% of survey respondents believed that T-low should be set at a ratio of 25%–49% of expiratory flow to PEFR, while 11% of survey respondents believed that T-low should be set at 50%–75% of the ratio of expiratory flow to PEFR. The ratio of the end-expiratory flow (EEF) to the PEFR was 0.75, and the resulting T-low (0.5 s) was sufficient to stabilize the alveoli. Therefore, T-low settings generally require EEF/PEFR ≥ 75%. Should there be significant CO_2_ retention during ventilation, T-low can be prolonged appropriately; however, it is essential to ensure that the EEF/PEFR > 50%. Approximately 40% of survey respondents felt that the maximum permissible TV range should be between 4 and 6 ml/kg. Previous studies have noted (30) that the TV in APRV mode is indirectly controlled by adjusting the T-low to maintain the TV between 4 and 6 ml/kg. T-low should also be rationally adjusted to obtain an adequate TV while the lungs are reopened. Approximately 43% of the survey respondents felt that P-high allowed a maximum setting range of 30 cmH_2_O. It is recommended that P-high be maintained at levels below 35 cmH_2_O to reduce trans-alveolar pressure and the risk of lung injury. A total of 74% of survey respondents felt that pressure support should be used during spontaneous breathing in the APRV mode of ventilation. In modern ventilator technology, APRV mode incorporates adaptive pressure support features. T-low plays a key role in controlling the end-expiratory lung volume and preventing airway collapse. However, when pressure support is integrated into the APRV mode, T-low spontaneously adjusts to the patient’s respiratory needs. While this approach improves ventilatory efficiency, it sometimes leads to large fluctuations in TV, which may be detrimental to the stabilization of lung volume and preservation of alveolar function.

Approximately 37.7% of the survey respondents believed that P-high should be gradually reduced to reach a target of 20 cmH_2_O when preparing for weaning in patients with ARDS using the APRV mode of ventilation, while 38.5% of the survey respondents believed that T-high should be gradually increased up to 10 s, which is consistent with previous studies (31). As the patient’s lungs recovered, the APRV parameters decreased with adjustments to P-high and T-high, gradually transitioning to CPAP in a process known as “drop and stretch.” It has been shown (17) that for every 1 cmH_2_O decrease in P-High, T-High increases by 0.5 s. Guidelines recommend (32) a simultaneous decrease in P-high by a gradient of 2 cmH_2_O and prolongation of T-high by a gradient of 0.5–1 s. During this time, the mean airway pressure does not change significantly, eventually transitioning to CPAP. In addition, 74.5% of survey respondents noted that patients with ARDS must meet certain criteria before switching to a CPAP mode, including FiO_2_ ≤ 40%, P-high ≤ 10 cmH_2_O, and T-high ≥ 10 s.

Our findings showed that the three most common factors hindering the use of APRV modalities include a lack of guidelines or standardized protocols for their use (56.5%), insufficient training (57.3%), and unawareness of APRV modalities (45.2%). Usually, APRV is not considered the preferred or primary mode of ventilation. Instead, it is often used as an alternative ventilation strategy when the traditional modes of conventional mechanical ventilation are not applicable or achievable. APRV requires careful, precise, and continuous titration of ventilator settings by clinicians, which adds to the technical requirements. Currently, there is a lack of consensus on the use of APRV and a lack of clear evidence that APRV improves clinical outcomes. Clearer evidence and more standardized ventilator strategies are needed if the widespread use of APRV as the primary mode of ventilation for patients with ARDS is required (1).

## 6 Strengths and limitations

Our study is the first to assess the awareness and practice of APRV mode in patients with ARDS among nurses in China. The survey was conducted at multiple centers and included extensive and comprehensive data. However, there are some limitations. First, generalizability is limited to Sichuan Province; caution is warranted when extrapolating findings to other Chinese regions with differing resource profiles. Second, voluntary online recruitment likely caused self-selection and non-response biases, potentially overrepresenting staff interested in mechanical ventilation and leading to an overestimation of APRV awareness relative to the broader ICU workforce. Third, the cross-sectional design precluded exploration of causal relationships or deeper drivers of low APRV awareness; future qualitative studies are needed to elucidate the root causes of limited APRV uptake. Fourth, while the questionnaire underwent expert validation and pilot testing, formal psychometric evaluation and a composite knowledge scoring framework were absent. This limits the ability to quantify competency levels or assess internal consistency. Finally, despite a sufficient sample size, analyses were restricted to descriptive statistics. Although inferential tests were feasible, the absence of a validated composite knowledge score precluded regression modeling. Furthermore, heterogeneous response options across parameter settings risked Type I errors from multiple comparisons in this exploratory context. Future studies utilizing psychometrically validated instruments are needed to quantify competency and identify predictors of APRV competence.

## 7 Conclusion

Considerable variability in APRV knowledge and clinical practice was observed among ICU nurses and respiratory therapists in Sichuan Province, China. The findings highlight a scarcity of comprehensive, systematic APRV training and a lack of consensus regarding parameter settings. The absence of clinical guidelines or standardized protocols emerged as the most frequently cited barrier to APRV implementation. These results underscore the urgent need for targeted educational initiatives, the development of evidence-based application standards, and the establishment of standardized management protocols to optimize the use of APRV in patients with ARDS.

## Data Availability

The original contributions presented in this study are included in this article/supplementary material, further inquiries can be directed to the corresponding authors.
